# Artificial intelligence-driven discovery of bioactive peptides: Computational approaches and future perspectives

**DOI:** 10.1016/j.abiote.2025.100014

**Published:** 2025-12-03

**Authors:** Xu Liu, Feifei Guan, Huiying Luo, Bin Yao, Jian Tian

**Affiliations:** Institute of Animal Science, Chinese Academy of Agricultural Sciences, Beijing, 100193, China

**Keywords:** Bioactive peptides, Antimicrobial peptides, Antioxidant peptides, Anti-inflammatory peptides, Multifunctional peptides, Artificial intelligence

## Abstract

Bioactive peptides, defined as amino acid chains exhibiting diverse biological functions such as antimicrobial, antioxidant, and anti-inflammatory activities, are primarily generated through protein digestion methods including enzymatic hydrolysis, physical processing techniques and controlled microbial fermentation. Conventional discovery techniques that rely on multi-stage separation processes, such as enzymatic digestion, ultrafiltration, ion-exchange chromatography, gel filtration chromatography, and reverse-phase high-performance liquid chromatography (RP-HPLC) inherently demand substantial laboratory resources and extended timeframes. To address these limitations, artificial intelligence (AI)-driven approaches have emerged as transformative discovery platforms. These computational pipelines systematically execute six critical phases: comprehensive data acquisition and curation, advanced feature engineering utilizing physicochemical descriptors, machine learning model construction using algorithms, iterative model training incorporating hyperparameter optimization, rigorous validation against benchmark datasets, and high-throughput bioactive peptide prediction. This comprehensive review critically evaluates recent AI applications across four key bioactive peptide categories including antimicrobial peptides, antioxidant peptides, anti-inflammatory peptides, and multifunctional variants. Furthermore, it proposes integrated enhancement strategies such as classifying peptides via their functional mechanism or using database-independent modeling approaches. Additionally, based on AI methods, scenario-specific peptide customization and prediction of bioactivity in digested proteomes are anticipated to be achieved in the future.

## 1 Introduction

Proteins serve as fundamental building blocks of life and play a role in virtually every biological process. Chemically, they are composed of amino acids, and numerous studies have shown that their functions are not limited to their complete form but also extend to smaller peptide segments, each contributing uniquely to various physiological processes [1]. For now, Over 5,000 bioactive peptides were record in the “BIOPEP-UWM” website with functions like anti-oxidative, antithrombotic, anti-hypertensive, anti-microbial, immunomodulatory, and infrequently, multifunctional activity and *etc.* [2]. The biological mechanisms of various type bioactive peptides were still not adequately studied. Bioactive peptides are widespread. Many foods or potential foods were analyzed for the discovery of bioactive peptides. The most typical foods are those with abundant protein content, such as milk, soybean and so on[[3], [4], [5], [6]]. In recent years, driven by deeper research, some peptide identification work has focused on unusual foods, such as crocodile meat, or those primarily used as seasonings, like garlic [7,8]. Moreover, with computer science assistance, analysis is not confined to readily available samples but also extends to samples that are difficult to obtain with traditional methods, such as bioactive peptides from the metagenome [9]. A much broader analysis provides a comprehensive perspective of the natural distribution of bioactive peptides, and it demonstrates that the sources of bioactive peptides are broad and diverse [10].

The bioactive peptide identification and discovery works are attractive and many methods has been developed. Traditional bioactive peptide discovery methods were depended on separated skills including ultrafiltration, ion exchange chromatography, gel filtration chromatography and reverse high performance liquid chromatography [11]. During the separation process, bioactivity screening was conducted to identify the fractions that contains the target peptides. While these methods can effectively detect bioactive peptides and determine their sequences using LC-MS, they are often labor-intensive and time-consuming.

To accelerate the discovery of bioactive peptides, computer-assisted methods such as virtual screening and molecular dynamics simulations have been employed [10,12]. These techniques enhance the identification of distinctive features in bioactive peptides. However, due to the inherent complexity of bioactive peptides, these methods alone are not sufficient to fully address all challenges in their discovery. In 1956, John McCarthy coined the term “Artificial Intelligence” to describe machines that can make decisions like humans [13]. Since then, a vast array of computer algorithms has rapidly emerged in this field. In basic, Artificial Intelligence (AI) can be defined by the functions it achieves, constituting a toolkit of strategies that integrates multi-dimensional approaches. Until now, machine learning including deep learning is an important branch in AI field which was also used in bioactive peptides research.

This paper presents an overview of AI workflow fundamentals and commonly used methods, along with various sources of bioactive peptides and their discovery approaches. Particular emphasis is placed on antimicrobial peptides (AMPs), antioxidant peptides (AOPs), anti-inflammatory peptides (AIPs), other attractive bioactive peptides and multifunctional peptides. Potential future developments and applications are discussed in the final section.

## 2 How AI works

At present, artificial intelligence (AI) has become one of the key focal points in academic research. In essence, AI serves as a powerful tool for analyzing and identifying the interrelationships among various entities. Building on this concept, the AI-driven bioactive peptide discovery and design process is outlined as two types. One is prediction model and another is *de novo* design.

**Fig. 1:**
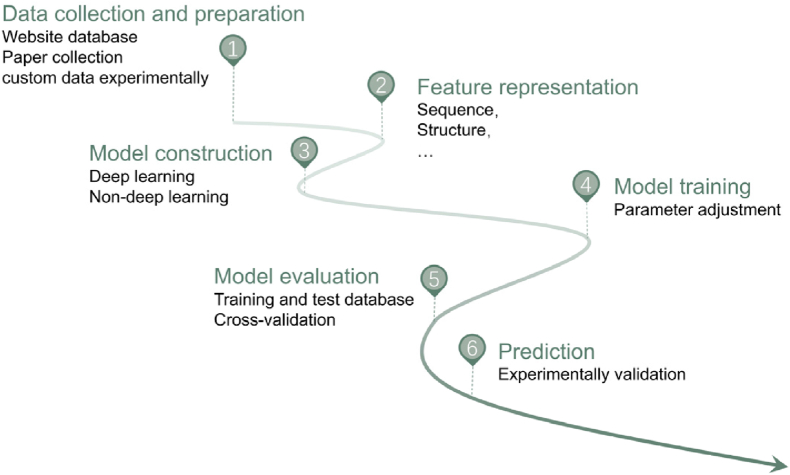
The workflow of AI assistant peptides prediction. The six-step workflow for AI-driven bioactive peptide discovery comprises: data collection and preparation, feature representation, model construction, evaluation and prediction.

For the prediction model construction, the major goal of the project should be first determined. Generally, AI-driven prediction progress includes two major types: classification model and regression model [10], which respectively address the questions “What is it?” and “How active is it?”. Next, the whole process includes the six steps: data collection and preparation, feature representation, machine learning/deep learning model construction, model training, evaluation and prediction (Fig. 1). The key to data preparation lies in expanding the overall data pool while maintaining data quality. Sometimes, online databases are not adequately providing the required amount of data. To achieve accurate prediction results, data collection from the literature, data deduplication, and reannotation of peptide details may be necessary. For peptides exhibiting novel functions or when existing datasets are limited, experimental data generation is essential [14]. The feature representation step converts complex biological details of a peptide, such as its sequence, structure and other properties, into raw data that machines can comprehend [15,16]. Furthermore, the field is witnessing a paradigm shift from manual feature engineering towards automated, data-driven representation learning. This is largely driven by the adoption of pretrained protein language models (pLMs), such as ESM and ProtBERT [17,18]. Unlike traditional descriptors defined by human experts, these models are self-supervised on millions of diverse sequences, enabling them to learn fundamental principles of protein biochemistry, evolutionary constraints, and even aspects of structural stability directly from data. The resulting context-aware embeddings provide a rich, information-dense starting point for models, often revealing functionally relevant patterns that are not captured by manual features. This approach has established a new state-of-the-art, providing a powerful and generalizable foundation for quantitative prediction tasks. Beyond single-activity prediction, AI models are now increasingly applied to the simultaneous prediction of multiple bioactive properties (multi-task learning) to identify multifunctional peptides.

The construction of machine learning models is a crucial step in building AI-driven predictive methods for bioactive peptides. For machine learning model construction, there are two major strategies, non-deep learning and deep learning. Support vector machines (SVM) and random forest (RF) are popular non-deep learning methods in bio model construction. The main goal of SVM is to locate a hyperplane that maximizes the margin between the positive and negative samples [19]. This approach effectively reduces misclassifications and improves the model's robustness. The RF algorithm is founded on the principles of random sampling and the construction of multiple decision trees. Each individual decision tree can uncover specific relationships within the data, and the collective predictions or insights from all trees are aggregated to produce the comprehensive result of the RF model [20]. Unlike random forests, eXtreme Gradient Boosting (XGBoost) and Light Gradient Boosting Machine (LightGBM) rely on gradient boosting, sequentially building trees to refine predictions [21]. Compared to non-deep learning approaches, deep learning models eliminate the need for manual feature engineering by automatically extracting task-specific high-level representations from raw peptide sequences, leading to more efficient development of predictive models [22]. Common deep learning model involved in bioactive peptides discovery included: Convolutional Neural Networks (CNN), Recurrent Neural Networks (RNN), Graph Attention Network (GAT) and so on. While CNNs provide an effective framework for hierarchical feature extraction through sliding-window convolutions and weight-sharing, their localized operations inherently struggle to capture long-range dependencies in data [23]. Long Short-Term Memory (LSTM) networks are an advanced variant of RNNs, designed with gating mechanisms to effectively model long-range dependencies in sequences [4]. Via attention mechanism, GAT achieved high performance in graph feature extraction [24]. Particularly, Transformer-based models, leveraging self-attention mechanisms, have emerged as powerful architectures for capturing complex, long-range dependencies within peptide sequences, setting new benchmarks in predictive accuracy [25].

Next, model training functions as a meticulously engineered optimization process driven by data pipelines, regularization techniques, and monitoring systems, where parameter optimization serves as the core mechanism. For model evaluation, an independent test set is essential. Model evaluation employs standard metrics such as AUC-ROC and F1-score for classification, and RMSE and Pearson's r for regression. Cross-validation is critical to ensure generalizability. The field is now advancing beyond these standard frameworks. Multi-task learning, which trains a single model to predict multiple activities simultaneously, is gaining traction for its efficiency and ability to model peptide polypharmacology. Furthermore, geometric deep learning approaches are being developed to directly leverage 3D structural information of peptides for predicting interactions and bioactivity. Once rigorously trained and validated, the model predicts properties of novel bioactive peptides or identifies new candidates. To ensure activity, rigorous experimental validation must follow. Notably, existing AI tools in bioactive peptide discovery frequently achieve strong results (Table 2).

**Table 1:** Bioactive peptide related database.

Type	Name	Description	Website	Reference
Bioactivity peptides	bioPEP-UWM	Focused on food source bioactive peptide and protein, including 5362 peptides.	https://biochemia.uwm.edu.pl/biopep/start_biopep.php	[2]
	DFDB	DFBP (Database of food-derived bioactive peptides) collected 7058 entries of food-derived bioactive peptides, 21,249 entries of food-derived proteins	http://www.cqudfbp.net/index.jsp	[6]
Antimicrobial peptides	APD3	the Antimicrobial Peptide Database (APD) contains 5099 peptides, including 3306 natural antimicrobial peptides (AMPs), 1299 synthetic, and 231 predicted AMPs.	http://aps. unmc.edu/AP/	[37]
	dbAMP	Encompassing 33,065 AMPs and 2453 antimicrobial proteins from 3534 organisms	https://awi.cuhk.edu.cn/dbAMP/	[38]
	DRAMP 4.0	DRAMP4.0 contains 30,260 entries, consisting of 11,612 general entries, 17,886 patent entries, 96 clinical entries, 377 stapled entries, 110 stability data and 179 expanded entries.	http://dramp.cpu-bioinfor. org/	[34]
	DBAASP	DBAASP provides users with information on the detailed structure (chemical, 3D) and antimicrobial activity of peptides against particular target species. The database contains information on ribosomal, non-ribosomal, and synthetic peptides that show antimicrobial activity as Monomers, Multimers, and Multi-Peptides.	https://dbaasp.org/home	[36]
	DADP	Database of Anuran Defense Peptides. It currently contains 2571 entries with a total of 1923 different bioactive sequences, of which 921 peptides have the minimum inhibiting concentration (MIC) against at least one microorganism	http://split4.pmfst.hr/dadp	[35]
	CAMP_R4_	Natural AMPs: 11,827 Synthetic AMPs: 12,416	https://camp.bicnirrh.res.in/	[33]
	AntiTbPdb	AntiTbPdb is a database of experimentally verified anti-tubercular or anti-mycobacterial peptides. AntiTbPdb contains around 1010 entries extracted from research papers and patents.	https://webs.iiitd.edu.in/raghava/antitbpdb/	[39]
Antioxidant peptides	AODB	AODB stored 56,666 small molecules tested for antioxidant activity, 1480 antioxidant peptides, and 998 antioxidant proteins, as well as their relevant information.	https://aodb.idruglab.cn/	[65]

**Table 2:** AI methods applied in bioactive peptides.

Type	Name/Case	Model classification	Data source	Dataset split strategy and leakage prevention			Algorithm	Performance	References
				Training/Validation set split strategy	Training/Test set split strategy	Key leakage prevention			
Antimicrobial	Macrel/Genome	Classification Model	ADP3, CAMP_R3_, LAMP	Out-of-Bag Estimation Training Set: 3268 positive sequences 165,138 negative sequences Ratio: ∼1:50	Training Set: 1197 positive sequences Varied number of negative sequences Test Set: 500 positive sequences 500 negative sequences	CD-HIT (<80 %)	RF	Sn = 0.90, Sp 0.998 ACC 0.95 MCC 0.90	[45]
	AMPlify/against WHO priority pathogens	Classification Model	APD3 DADP	5-Fold Cross-Validation 1669 positives sequences 1669 negatives sequences	Random split (80/20) Training set 1669 positives sequences 1669 negatives sequences Test Set 418 positives sequences 417 negatives	No sequence-similarity check between training and test sets.	Bi-LSTM Attention	Sn 0.9293 Sp 0.9449 ACC 0.9371 F1 0.9366 AUC 0.9837	[40]
	sAMPpred-GAT	Classification Model	SATPdb, ADAM, AMPfun, APD3, CAMP, LAMP, DRAMP, dbAMP	Internal Random (80/20) 5536 positives sequences 5536 negatives sequences	Strict Isolation Independent external test sets 3719 positives sequences 3400 negatives sequences	CD-HIT (Positives <70 % Negatives <40 %)	GAT	Sn 0.53 Sp 0.9 ACC 0.715 MCC 0.464 AUC 0.777	[41]
	AMPpred-MFA	Classification Model	CAMP_R3_ APD3 DRAMP 2.0	5 trials with 85/15 train-test split & 80/20 train-validation split Training/Validation Pool (85 %) ∼3463 positive sequences ∼3463 negative sequences	Independent Test Set (15 %) ∼611 positive sequences ∼611 negative sequences	CD-HIT (positives ≤90 %, negatives ≤40 %) Test set isolated before undersampling	LSTM CNN	Sn 0.946 ± 0.01 SP 0.941 ± 0.006 ACC 0.943 ± 0.004 MCC 0.866 ± 0.008 AUC 0.983 ± 0.002	[23]
	DeepMAMP/milk protein	Classification Model	DBAASP	Not Applicable	Random Split (80/20) 2450 positives sequences 2508 negatives sequences	No sequence-similarity check between train and test sets Independent, source-segregated validation set (MDdata)	LightGBM LSTM Attention	ACC 0.814 Pre 0.934 Sn 0.849	[4]
	MBC-Attention/Against *E. coli*	Regression Model	DBAASP	Repeated Random Subsampling (90/10) Total 3536 sequences	Random split (≈90/10) Training Set 3536 sequences Test Set 393 sequences	No sequence-similarity check	CNN Attention	Pearson's r 0.775 ± 0.014 CCC 0.738 ± 0.015 R^2^ 0.588 ± 0.005 MAE 0.402 ± 0.006 MSE 0.284 ± 0.003 RMSE 0.533 ± 0.002	[44]
	MODAN/non-proteinogenic amino acids	Generation Model	In-house dataset	10-Fold Cross-Validation Used on the initial dataset (82 peptides) for feature engineering and surrogate model selection, not for the final generative loop.	Iterative Experimental Validation No static test set. The model was evaluated through two rounds of experimental feedback.	Temporal Segregation/Iterative Hold-out	Gaussian Process Regression	Pearson's r 0.7–0.88	[42]
	ProteoGPT and its submodels/against multidrug-resistant bacteria	Generation Model	APD3, DBAASP, DRAMP, CAMP	Random Splitting Training: validation: test = 6:2:2 16,062 positive sequences 16,549 negative sequences		CD-HIT (<70 %)	Transformer	AMPSorter: AUC 0.99 (test set) AUC 0.97 (benchmarking set) Pre 0.9643 BioToxiPept: AUPRC 0.92, AMPGenix: recognition rate (76.1 % identified as AMPs)	[48]
Antioxidant	AnOxPePred	Classification Model Regression Model	BIOPEP-UWM Papers	5-Fold nested Cross-Validation 685 AOP sequences 606 free radical scavengers 11 CHEL chelators both 70; non-antioxidant 717 sequences 217 experimentally-validated 500 random		Nested Cross-Validation; Homology-Controlled Partitioning Global Dataset Deduplication; Random Negative Sampling; Independent Experimental Validation	RF SVM CNN	Scavenging free radical AUC 0.79 F1 0.72 MCC 0.48 Chelating metal ion AUC 0.60 F1 0.25 MCC 0.28	[68]
	AnOxPP	Classification Model	DFBP BIOPEP-UWM	Random Splitting (80/20) 1060 positive sequences 1060 negative sequences	72 highly active antioxidant peptides that have no intersection with the training and validation datasets collected from literatures	Independent External Validation Sets Homology Reduction in Negative Samples No sequence-similarity check between train and test sets	Bi-LSTM	Sn 0.9434 Sp 0.9906 ACC 0.9670 MCC 0.9350 Pre 0.9901	[69]
	AOPxSVM	Classification Model	DFBP BIOPEP-UWM PlantPepDB	Random Splitting (80/20) 1511 positive sequences 1511 negative sequences	75 highly active antioxidant peptides that have no intersection with the training and validation datasets collected from literatures	Independent External Validation Sets Homology Reduction in Negative Samples No sequence-similarity check between train and test sets	SVM	Sn 0.9200 Sp 0.9467 ACC 0.9333 MCC 0.8670 Pre 0.9452 F1 0.9324	[67]
Anti-inflammatory	antiInflam	Classification Model	IEDB	Random Splitting (80/20) 863 positive sequences 1262 negative sequences	No independent test dataset	No sequence-similarity check between train and test sets No independent test dataset	SVM	Sn 0.7861 Sp 0.6736 ACC 0.72 MCC 0.45	[84]
	AIPpred	Classification Model	IEDB	5-Fold Cross-Validation 1258 positive sequences 1887 negative sequences	Random Splitting (8:2) 1678 positive sequences 2516 negative sequences	CD-HIT (<80 %)	RF	Sn 0.741 Sp 0.746 ACC 0.744 MCC 0.479 AUC 0.814	[85]
	preAIP	Classification Model	Same as AIPpred	5-Fold Cross-Validation 1258 positive sequences 1887 negative sequences	Random Splitting (80/20) 1678 positive sequences 2516 negative sequences	CD-HIT (<80 %/60 %)	RF	Sn 0.709 Sp 0.806 Ac 0.767 MCC 0.508 AUC 0.833	[15]
	iAIPs	Classification Model	Same as AIPpred	5-Fold Cross-Validation 1258 positive sequences 1887 negative sequences	Random Splitting (80/20) 1678 positive sequences 2516 negative sequences	CD-HIT (<80 %)	RF	Sn 0.567 Sp 0.874 ACC 0.751 MCC 0.471 AUC 0.822	[86]
	AIPPT	Classification Model	IEDB	Random Splitting training dataset 1511 positive sequences 1511 negative sequences, validation dataset 168 positive sequences 168 negative samples.	Independent test dataset1 187 positive sequences 187 negative sequences Independent test dataset1 420 positive sequences 629 negative sequences	CD-HIT (<80 %)	RF XGBoost LightGBM Logistic regression	Sn 0.800 Sp 0.801 ACC 0.800 MCC 0.593 AUC 0.787	[87]
	IF-AIP	Classification Model	Combination of iAIP andantiInflam	Repeated stratified 5-fold cross-validation Training dataset 1451 positives sequences 2339 negatives sequences	Independent test dataset 1 420 positive sequences 629 negative sequences Independent test dataset 2 173 positive sequences 253 negative sequences	CD-HIT(<90 %) New experimental data	RF extra tree classifier XGBoost LightGBM CatBoost	Independent test 1 Sn 0.690 Sp 0.874 ACC 0.800 MCC 0.579 AUC 0.873 Independent test 2 Sn 0.803 Sp 0.742 ACC 0.777 MCC 0.536 AUC 0.871	[88]
	DeepAIP	Classification Model	Combination of PreAIP, AIPpred, andIF-AIP	Random Splitting 1365 positive sequences 2218 negative sequences	Random split (80/20) Training Set 1365 AIPs 2218 Non-AIPs Test Set 342 AIPs 555 Non-AIPs	CD-HIT (<90 %) Training-Only Augmentation Training-Only Feature Engineering: PCA transformation	Pre-trained protein language model feature extraction Contextual Self-Attention Model CNN	Sn 0.8070 Sp 0.8523 ACC 0.8350 MCC 0.6539 AUC 0.89	[89]
Multifunctional	MLBP	Multi-label classification model	Collect from PreAIP, mAHTPred, BioDADPep, antiCP 2.0, and AMPfun	5-Fold Cross-Validation on the training set. The training set contained 5900 samples (516 ACPs + 411 ADPs + 694 AHPs + 1342 AIPs + 1937 AMPs). The validation role was served by the rotating folds within this set.	Random split (80/20) Training Set 5900 samples (80 %) Test Set 1225 samples (20 %) (130 ACPs + 103 ADPs + 174 AHPs + 336 AIPs + 482 AMPs)	CD-HIT (<90 %)	Multi-label Learning Embedding layers CNN BiGRU Fully Connected Layer Sigmoid Activation Function Binary Cross-Entropy Loss Thresholding	Pre 0.709 ± 0.006 Coverage 0.717 ± 0.006 ACC 0.708 ± 0.006 Absolute True 0.699 ± 0.006 Absolute false 0.106 ± 0.003	[107]
	MMDB	Multi-label classification model	Same as MLBP	Random split (80/20) Total Peptides: 6440 Training Set 5152 peptides Validation Set 1288 peptides	No independent test dataset	CD-HIT (<90 %)	Multimodal Data Fusion Embedding Layer Multi-scale Dilated Convolution Bi-LSTM CNN Fully Connected Layer Sigmoid Activation Function: Binary Cross-Entropy Loss Thresholding	Pre 0.734 Coverage 0.765 ACC 0.733 Absolute True 0.701 Absolute false 0.102	[106]
	DLFea4AMPGen	Generation model	Twenty BPs datasets for eighteen different bioactivities were collected [109]	10-Fold Cross-Validation	Follow the research method [109]	10-Fold Cross-Validation avoid overfit	Transformer MP-BERT SHAP K-means	AUC 0.961	[108]

ACC: accuracy; AUC: Area Under the Receiver Operating Characteristic Curve; BiGRU: Bidirectional Gated Recurrent Units; Bi-LSTM: bidirectional long short-term memory; CatBoost: Categorical Boosting; CCC: concordance correlation coefficient; CNN: Convolutional Neural Networks; GAT: Graph Attention Network; LightGBM: Light Gradient Boosting Machine; LSTM: Long Short-Term Memory; MAE: mean absolute error; MCC: Matthews Correlation Coefficient; MSE: mean squared error; Pre: precision; RF: Random Forest; RMSE: root mean squared error; Sn: Sensitivity; Sp: Specificity; SVM: Support Vector Machines; XGBoost: eXtreme Gradient Boosting.

In recent years, *de novo* bioactive peptide design has become increasingly attractive. Instead of aiming to explain the properties of natural peptides, this method makes it possible to overcome the limited diversity of natural amino acid sequences and develop peptides with superior activity. In short, this method addresses the question: “How can we obtain a new bioactive peptide?” The workflow for *de novo* bioactive peptide design is similar to that of a predictive model, with one key difference: a generation step precedes the prediction step. In this generation step, candidate sequences are sampled from a trained generative model that has learned the underlying distribution of functional peptides. For constructing a virtual peptide library, effective generative approaches include Generative Adversarial Networks (GANs), Variational Auto-Encoders (VAEs), and Transformer models. Rule-based search algorithms, such as Genetic Algorithms (GA) and Monte Carlo Tree Search (MCTS), are also widely employed [26]. The subsequent prediction step then estimates their relative bioactivity. Furthermore, in modern generative models, the generation and prediction steps are closely integrated to enable efficient production of high-efficacy sequences. Here, reinforcement learning is increasingly used to guide the generative process towards sequences that optimize multiple desired properties simultaneously, such as potency, stability, and low toxicity.

## 3 Antimicrobial peptides

**Fig. 2:**
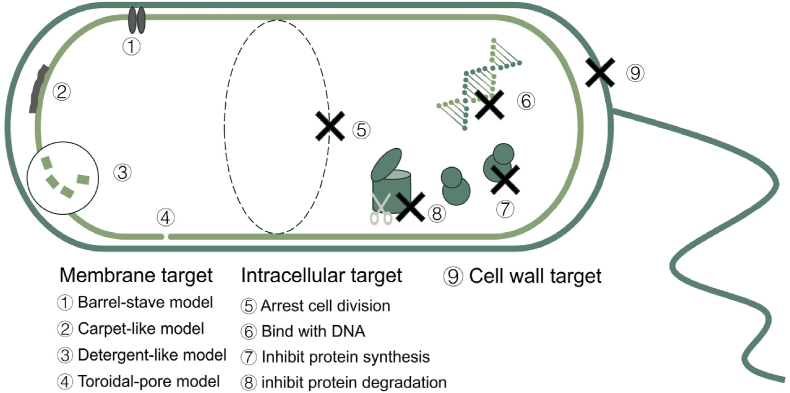
The mechanism of antimicrobial peptides. Antimicrobial peptides target microbes at multiple locations, ranging from external structures to internal components, such as the cell wall, membrane, and intracellular elements.

Antimicrobial peptides (AMP) derive their name from their functional properties. They can be categorized into diverse types based on the target organisms they act against, such as antibacterial, antifungal, antiviral, and other peptides [27]. Generally, AMPs are 10–100 amino acids in length and most with a molecular weight of less than 10 kDa [28]. Beside the different object, the mechanisms of one type were also various, including the target the cell wall, cytomembrane or inside the cell (Fig. 2). Furthermore, the target cytomembrane type also works in different manners. There are four proposed models for membrane-targeting AMPs, including Barrel-stave model, Toroidal-pore model, Carpet-like model, Detergent-like model. For the intracellular targeting, it was more complex for the target including nucleic acid targeting, protein targeting, protease targeting, and others [29]. For each target, AMPs operate via distinct mechanisms. For example, membrane-targeting peptides are typically enriched in both hydrophobic and positively charged amino acid residues. However, the mechanisms of AMP action against the cell wall and intracellular targets remain largely characterized on a case-by-case basis and require deeper investigation to elucidate any potential common or unifying principles [30,31]. In specific cases, AMP coding genes could also improve channel catfish antidisease resistance [32].

To accelerate AMP discovery and characterization, diverse dedicated databases have been developed (Table 1). Some AMP databases, such as APD3, dbAMP, DRAMP, DBAASP, and CAMP_R4_, focus on comprehensive collections, while others target specific AMP types, like those against particular strains (e.g., AntiTbPdb) or specific sources (e.g., DADP, which focuses on peptides derived from invertebrates) [[33], [34], [35], [36], [37], [38]]. In detail, APD3 mainly focuses on natural antimicrobial peptides, which occupy over 60 % of its total data [37]. Besides, it also lists AMPs with other functions like anti-cancer or anti-inflammatory. dbAMP collects not only AMPs but also over 2000 antimicrobial proteins [38]. DRAMP serves as a major repository, extensively curating peptides with detailed activity annotations against various pathogens [34]. DBAASP specializes in non-natural peptides and AMP 3D structures, providing detailed structural information [36]. Beyond collecting AMP data, CAMP_R4_ also supports AMP prediction [33]. AntiTbPdb serves as a specialized database for anti-tubercular and anti-mycobacterial peptides [39].

Base on the constructed databases, there are growing research focused on AMP discovery, creation and application driven by AI approaches. These methods’ name, model types, data source, dataset split strategy and leakage prevention, algorithm and performance were all collected in Table 2. In 2022, amplify method was developed for discovery of AMPs from bullfrog genome for use against WHO priority pathogens [40]. Although the approach yielded positive outcomes, the model construction lacked refinement, particularly in constraining sequence similarity between the training and test datasets. DeepMAMP, a combination model with LightGBM, LSTM and attention mechanism, has successfully predict 322 antimicrobial peptides from human milk [4]. Similarly, AMPs in the gut microbiome are also a prominent research focus, with 181 such peptides experimentally identified [9]. While accumulating data, the AMP structure was also considered for AMP property extraction [41]. Moreover, some research not only focus on antimicrobial peptides composed with 20 proteinogenic amino acids, the non-proteinogenic or modified amino acid were also analyzed to improve antimicrobial activity and the ability to penetrate cells [42]. Recent advances focus on enhancing predictive precision for specific biological targets and quantitative endpoints such as minimum inhibitory concentrations (MIC) [43,44]. Looking ahead, mechanism-driven classification of peptides could unlock precisely tailored therapeutic applications. Complementing this, molecular profiling of pathogens may reveal targetable vulnerabilities, enabling rational selection of antimicrobial peptides with unprecedented precision. In the context of AMP sources, Macrel was developed to discover AMPs directly from genomic data [45]. Leveraging this approach, the AMPSphere project successfully harnessed it to identify nearly 1 million AMP candidates from 63,410 metagenomes and 87,920 microbial genomes [46].

Research into the *de novo* design of AMPs has been growing. Fundamentally, these computationally generated AMPs can leverage the advantages of multiple natural AMPs, resulting in improved efficacy. Huang et al. reported a method to discover AMPs from a huge virtual peptide library, which showed therapeutic efficacy comparable to penicillin in mice bacterial pneumonia model [47]. While the pipeline is composed of discriminative models, it mimics the exploratory scope of a generative model. A key difference lies in its library construction, which is based on exhaustive enumeration of sequences rather than learned generation. Commonly, the generation models utilize pre-trained protein language models to systematically capture critical peptide features for constructing virtual peptide libraries. This strategy could avoid the combinatorial explosion resulting from random combinations. In detail, AMPGenix is a generative model built upon ProteoGPT, a pre-trained protein large language model (LLM). ProteoGPT comprises more than 124 million parameters and served as the foundation for fine-tuning to develop AMPGenix [48]. Using this model, 7,798 peptides were generated. After the removal of potentially toxic peptides, 42 were selected for experimental validation. Eight peptides showed antimicrobial activity, and three of them demonstrated broad-spectrum activity. Another case was also focused on to generate high-quality AMP sequences. Moreover, it showed a lower propensity for inducing resistance [48]. With technological advancement, more potent AMPs can be developed for therapeutic use.

## 4 Antioxidant peptides

Besides antimicrobial peptides, antioxidation peptides (AOP) is another type bioactive peptides attract people's attention. Oxidation is a normal process happened every time and everywhere in life exist or not exist space. Oxygen is fundamental to life; However, it is also highly reactive and can interact with many biomolecules within cells, leading to the generation of reactive oxygen species (ROS) [49]. ROS are a broad category of highly reactive oxidizing agents derived from molecular oxygen. Under normal conditions, the body's oxidant defense system, which includes antioxidant enzymes and agents, maintains a balance with ROS [50]. However, in many diseases such as diabetes, Alzheimer's disease, arthritis, heart disease, and cancer, this equilibrium is disrupted [51,52]. In such cases, antioxidant therapy is a common strategy treatment. Besides, antioxidant agents are also used in skin protection and anti-aging, as ROS can cause damage to DNA [53,54].

**Fig. 3:**
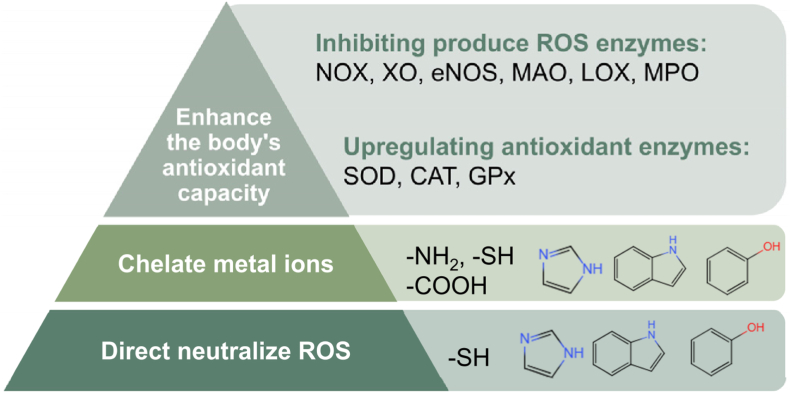
The mechanism of antioxidant peptides. Antioxidant peptides function through their inherent chemical properties (neutralizing ROS or chelating metal ions) or by stimulating the body's antioxidant capacity.

In recent years, antioxidant peptides have also emerged as a topic of growing interest. As degradation products of proteins, peptides have a wide range of sources. Diverse types of proteinases can react with thousands of proteins, thereby producing a variety of peptides with different functions. In terms of mechanism, antioxidant peptides may function at three distinct levels (Fig. 3). First, direct react with ROS. Some amino acids in antioxidant peptides contain antioxidant functional groups that can donate hydrogen atoms or electrons to neutralize ROS, such as the phenolic hydroxyl group, imidazolyl, and indolyl that can donate hydrogen atoms and sulfhydryl that can donate both hydrogen atoms and electrons [55]. Second, antioxidant peptides can chelate metal ions, such as Fe^2+^ and Cu^2+^, which helps prevent the generation of hydroxyl radicals OH^−^ from H_2_O_2_ [56]. The functional groups like amino group NH_3_^+^, carboxyl group COOH, sulfhydryl, imidazolyl, and indolyl in the peptides have strong affinities to metal ions, enabling effective chelation. Lastly, antioxidant peptides can enhance the body's antioxidant capacity by inhibiting enzymes that produce ROS and upregulating the expression of antioxidant enzymes [57]. For instance, they can inhibit the activity of enzymes such as reduced nicotinamide adenine dinucleotide phosphate oxidase (NOX), xanthine oxidase (XO), endothelial nitric oxide synthase (eNOS), monoamine oxidase (MAO), lipoxygenase (LOX), and myeloperoxidase (MPO), or downregulate their expression levels [[58], [59], [60], [61], [62], [63]]. Moreover, some antioxidant peptides can upregulate keap1-Nrf2 signaling path and activate the expression of downstream antioxidant enzymes, such as superoxide dismutase (SOD), catalase (CAT) and glutathione peroxidase (GPx) [64].

In the field of antioxidant peptides, the number of databases is much less than the antimicrobial peptides database. The reason may belong to the mechanism complex of antioxidant. The database AODB has collected antioxidant agent not only peptides, but also small molecules [65]. In the bioactive peptides database BIOPEP-UWM, also contained about 900 antioxidant peptides. There are also some AI assistant methods for antioxidant peptides discovery. In different protein source, *in silico* methods provided many antioxidant peptides. Moreover, some generative models were applied and provided artificial designed antioxidant peptides. Via combination of generation model and Density functional theory (DFT) calculations, 6 peptides exhibited antioxidant activity comparable with vitamin C [66]. AnOxPePred, AnOxPP and AOPxSVM are three AI-based antioxidant peptide prediction methods that used CNN, BiLSTM and SVM combined with LightGBM for model construction respectively[[67], [68], [69]]. AnOxPePred has collected AOP from the BIOPEP-UWM database and relevant literature, categorizing them as free radical scavengers or chelators based on their mechanisms of competing with ROS [68]. The limited performance of the chelator prediction may be attributable to the small dataset size. AnOxPP improved prediction performance using a BiLSTM deep learning model [69], and AOPxSVM achieved similar gains through feature engineering optimization [67]. In specific protein source antioxidant peptide discovery, AI were also used via simulated enzymatic digestion and peptides activity valuation. Relying on AI algorithm assistant, many antioxidant peptides were identified from various food or feed source such as chicken, eggshell membrane, squid skin protein, cottonseed protein, and rice bran protein [[70], [71], [72], [73], [74]].

## 5 Anti-inflammatory peptides

Unlike antimicrobial peptides or antioxidant peptides, anti-inflammatory peptides must function within the host's own highly complex inflammatory response network, which involves intricate crosstalk between multiple immune cells, signaling pathways, and mediators [75]. At its core, inflammation is a complex and indispensable physiological process activated in response to tissue injury or infectious agents. Beyond its core roles in pathogen elimination, debridement, and tissue repair, inflammation provides an essential alerting function—manifesting as pain and swelling—to drive avoidance of further injury and promote healing. However, excessive inflammation can cause prolonged pain and increase the risk of cancer [76]. As a result, anti-inflammatory therapy is necessary for many cases such as wounds, infection and autoimmunity disease.

To understand the mechanism of anti-inflammatory peptides, the inflammation process needs to described detailly. Typically, an inflammatory reaction involves four key steps: recognition, activation, signal release, and immune cell recruitment [77]. Once recognition of stimuli occurs, receptor activation triggers common signaling pathways including the nuclear factor kappa-B (NF-κB) and mitogen-activated protein kinase (MAPK). NF-κB signaling is indispensable for inflammation. Upon activation, the p50/p65 heterodimer translocates to the nucleus, where phosphorylation of p65 licenses the transcription of downstream inflammatory genes. Upon activation, this pathway significantly upregulates the expression of various pro-inflammatory mediators, including cytokines (e.g., TNF-α, IL-1β, IL-6, IL-12), adhesion molecules (e.g., ICAM-1, VCAM-1), inflammatory enzymes (e.g., iNOS, COX-2), and immunoreceptors (e.g., MHC). Although MAPK pathway is more related to cell cycle regulation, it could also unregulated inflammatory genes expression above via transcription factors such as AP-1 and ATF-2. Thus, the NF-κB and MAPK pathways function as parallel yet interconnected signaling cascades in the inflammatory response.

To counteract this, anti-inflammatory peptides modulate NF-κB through distinct mechanisms: inhibiting ligand-receptor binding, preventing p65 complex translocation, or suppressing p65 phosphorylation[[78], [79], [80], [81], [82]]. Anti-inflammatory peptides target the MAPK pathway through three principal mechanisms: inhibition of receptor binding, blockade of key enzyme phosphorylation, or suppression of transcription factor activation. Notably, some peptides exhibit context-dependent anti-inflammatory activity secondary to core antimicrobial/antioxidant functions. Their classification as “anti-inflammatory peptides” based solely on phenotypic effects contradicts mechanism-driven taxonomy, creating operational challenges for database harmonization.

To date, several AI-driven methods have been developed for predicting AIPs. Representative predictors include antiInflam, AIPpred, preAIP, iAIPs, AIPPT, IF-AIP and DeepAIP, all summarized in Table 2. In these methods, The Immune Epitope Database (IEDB) was used as a source of positive data. The IEDB catalogs published epitopes and their contextual experimental data in a freely searchable public resource, which gathers experimentally validated epitopes that trigger at least one anti-inflammatory cytokine (IL-10, IL-13, IL-22, IL-1RA, TGF-β, IFN-α/β) [83]. AntiInflam was an early AIP prediction tool based on machine learning [84]. Leveraging an enlarged dataset, more efficient feature extraction and optimized parameters, AIPpred achieved slightly improved performance [85]. For improving prediction performance, preAIP and iAIP applied more efficient feature coding methods [15,86]. In terms of algorithm improvement, AIPPT used a stacked ensemble model of LightGBM, RF, and XGBoost alongside logistic regression for data classification [87]. Similarly, the IF-AIP method employed a voting classifier to enhance performance [88]. DeepAIP employed a deep learning approach and demonstrated high accuracy by successfully identifying 17 experimentally confirmed AIPs (reported in existing database) that were not included in its training or test datasets, validating its ability to recognize anti-inflammatory activity [89]. By coupling refined feature extraction with deep learning, these tools have progressively improved accuracy. It is worth noting that all these mothered all used the positive data from IEDB database, which only focus on the epitope's immunoreaction and was not deeply related to the mechanism of AIP discussed in this study. Future efforts should integrate larger, high-quality datasets alongside more detailed molecular mechanisms to further enhance AIP prediction.

## 6 Other bioactive peptides

Furthermore, there are many other attractive bioactive peptides reported. Anti-hypertensive peptides (AHP) could reduce blood pressure via modulating the renin-angiotensin system (RAS) or enhancing eNOS pathway to increase nitric oxide (NO) levels within vascular walls and promote vasodilation [90]. In the discovery of AHP, Gradient Boosted Decision Trees (GBDT) have been used to screen bioactive peptides, while LLMs have been employed to optimize digestive enzyme combinations [91,92]. Anxiety disorder is a prevalent mental health issue globally. Fortunately, there were bioactive peptides discovered via AI-driven or traditional methods with neuroprotective effects, especially on cognitive dysfunction [93,94]. Moreover, a growing number of bioactive peptides are being discovered, such as anti-diabetic, immunomodulatory, anticancer and anti-aging peptides[[95], [96], [97], [98], [99]]. This expansion in target diversity illustrates the broadening scope of AI-driven peptide discovery. The field is no longer confined to a few classic activities but is rapidly diversifying to encompass a more holistic, systems-level approach against complex diseases.

## 7 Multifunctional peptides

The combination of multiple functional domains is a common organizational strategy in proteins, with over 67 % of proteins possessing more than one domain [100]. Given the significantly shorter sequence length of peptides, a key question arises: can they also exhibit diverse functions? Generally, two scenarios enable peptide multifunctionality. First, a single peptide may intrinsically harbor multiple mechanisms of action. For example, Temporin G possesses both antibacterial activity and the ability to block viral entry into cells [101]. Second, some peptides have a primary mechanism but demonstrate additional functions in specific contexts. An antioxidant peptide, for instance, may exhibit anti-inflammatory activity in an infection model. Distinguishing between these two types of multifunctional peptides is crucial for enhancing the quality of biological databases.

Up to now, there are two types of research focusing multifunctional peptides. First type is to explore a bioactive peptide exist another type activity. Another type is to create multifunctional peptides by combination different functional peptides together. Current research confirms that natural bioactive peptides embedding virtually any pairwise combination of well-defined functionalities, such as antihypertensive, anticancer, anti-inflammatory, antidiabetic, antimicrobial, and antioxidant activities. Although combination of various activities is a common phenomenon among peptides, most studies keep focusing on activities with synergistic effect such as antimicrobial, antioxidant and anti-inflammatory. Some research indicated that, the anti-inflammatory peptide often have the antimicrobial and antioxidant potential [75]. In the field of metabolic disease, antidiabetic and antithrombotic activities are usually considered together. For rational design of multifunctional peptides, a typical case was fusing the functional peptide with a cell penetrating peptide (CPP) or cell targeting peptide (CTP), which could avoid “off target” effect. Moreover, hybrid peptides were also applied in antimicrobial for improving specific pathogeny recognition or elevating antibacterial activity [102,103]. Furthermore, toxicity poses a critical barrier in bioactive peptide development. Consequently, the establishment of robust toxicity prediction models and the subsequent selection of low-toxicity candidates have become an integral step in the discovery pipeline [104].

For multifunctional peptide discovery, a straightforward method is to integrate predictions from several AI-based bioactive peptide discovery tools and take the intersection of their positive results [105]. Furthermore, deep learning methods were applied as multifunctional peptides classification, such as MLDP (Multi-Label deep learning approach for determining the multi-functionalities of Bioactive Peptides) and MMDB (Multimodal Dual-Branch) [106,107]. The MMDB model achieved better performance in predicting the multifunctionality of bioactive peptides by integrating multimodal data input and a dual-branch structure. It provides a more comprehensive description of peptides and learns richer functional information, thereby enhancing prediction accuracy. Additionally, its dual-branch structure makes the model more efficient, suitable for practical prediction tasks. DLFea4AMPGen efficiently generates high-quality AMP sequences by analyzing known AMPs with deep learning to identify key features. These features inform the construction of a diverse peptide library, which is refined to ensure stability and potential activity. The method accelerates AMP discovery and allows for experimental validation of generated sequences [108]. The designed AMP exhibited potent antimicrobial activity comparable to vancomycin, polymyxin B, or kanamycin. Building upon the UniDL4BioPep framework, DLFea4AMPGen strategy integrates explainable AI-derived features from multiple bioactivity models to enable the de novo design of multifunctional antimicrobial peptides [109]. Leveraging big data, future AI-driven methods will advance beyond multifunctional peptide prediction to efficiently design peptides engineered for complex scenarios — such as inflammatory bowel disease (IBD) treatment requiring integrated antioxidant and anti-inflammatory capabilities.

## 8 Conclusion and discussion

Bioactive peptides have emerged as a prominent research focus in recent years, demonstrating diverse bioactivities such as combating pathogens, maintaining metabolic homeostasis, and even including taste-active peptides that provide distinct flavors. Otherwise, bioactive peptides originate from a wide array of sources. These include multicellular organisms, marine organisms, plants, as well as proteins that have undergone physical, chemical, or biological treatment [110]. This review discusses antimicrobial peptides (AMPs), antioxidant peptides (AOPs), anti-inflammatory peptides (AIPs), and multifunctional peptides, along with their AI-driven discovery methodologies.

AI has advanced significantly in modeling complex relationships within big data, demonstrating notable success in bioactive peptide research. The computational methodologies have evolved from traditional machine learning (e.g., SVM, RF) to more sophisticated deep learning architectures, including Convolutional Neural Networks (CNNs) for local motif identification, Recurrent Neural Networks (RNNs/LSTMs) for sequence dependency modeling, and Transformer-based models that provide context-aware representations through self-attention mechanisms. However, in some cases, the prediction performance falls short of expectations. One reason is that classification based solely on peptide function, rather than deeper underlying mechanisms, can lead to misclassification or category overlap. The foundational issue resides in database inadequacy, manifesting as sparse specialized category data and inconsistent functional annotations. This data scarcity directly heightens the risk of model overfitting, which must be mitigated through strategies such as rigorous cross-validation and transfer learning from large protein language models (e.g., ESM, ProtBERT). These technical shortcomings converge with intrinsic biological complexity. Bioactive peptides are typically classified by their functional roles (e.g., antimicrobial, antioxidant). Yet peptides sharing a primary function often operate through entirely distinct mechanisms. For instance, antioxidant peptides function through diverse mechanisms, such as directly scavenging ROS or activating cellular antioxidant pathways. These distinct mechanisms highlight corresponding differences in peptide structure. This biological complexity underscores a critical challenge in AI interpretability: moving beyond “black-box” predictions to understand the rationale behind model outputs using Explainable AI (XAI) techniques is essential for gaining trustworthy and actionable biological insights. Moreover, the functional label of the peptides may encompass cross-functional cascades. For example, the anti-inflammatory effects of some peptides arise indirectly through antioxidant or antimicrobial activity. Based on these facts, the positive data or negative data in database were not strictly Binary classification. With technological development, data labels will become more specific and precise, significantly improving prediction performance in the future.

To date, significant efforts have focused on AI-driven discovery and production of bioactive peptides. Predominant methodologies follow a standardized pipeline: curated database construction, model training and validation, computational peptide screening, and experimental verification. Database quality serves as a critical determinant of model performance, making its enhancement a research priority. Furthermore, while database-independent modeling approaches show promise, developing robust evaluation frameworks represents an equally vital direction for advancing predictive accuracy [111].

Furthermore, a deeper understanding of bioactive peptide mechanisms is essential not only for constructing comprehensive databases but also for effectively matching peptides to specific application scenarios. Evaluating peptides solely based on unidimensional activity metrics (e.g., potency levels) is insufficient; comprehensive functional profiling is required to determine their optimal use cases. For instance, in antimicrobial applications, identifying target pathogens critically informs peptide selection efficacy. When targeting organisms lacking a conventional cell wall (e.g., Gram-negative bacteria), peptides acting primarily on peptidoglycan become unsuitable.

With access to high-quality databases and refined classification methods, AI could potentially enable scenario-specific peptide customization. Beyond simple multiple functional peptides fusion, this approach would offer an efficient and cost-effective strategy for de novo design of multifunctional peptides to address complex biological challenges. For instance, in pathogen-induced inflammation, engineered therapeutic candidates could concurrently deliver antimicrobial and anti-inflammatory effects, with potential augmentation by cell-penetrating capabilities to enhance targeting. Critically, simply fusing existing functional peptides may produce oversized hybrids that compromise synthetic feasibility and bioactivity while providing no guarantee of retained functionality or synergistic effects.

In real-world applications, proteins, rather than specific peptides, are typically used for more cost-effective implementation. Although microbial treatment or physical methods like high-voltage pulsed electric fields (HVPEF) can degrade proteins into bioactive peptides, enzymatic hydrolysis remains the conventional production approach [112]. Computational methods can simulate this process, and the resulting peptides can be screened using AI-driven bioactive peptide prediction tools. This integrated workflow provides an efficient approach for identifying proteins with high bioactive potential post-digestion. However, a critical challenge remains: the lack of a standardized metric to quantitatively compare the functional efficacy across different protein samples and digestion methods. Developing such an evaluation framework is essential for optimizing bioactive peptide yields. For instance, even highly bioactive peptides derived from low-abundance source proteins may yield insufficient bioactive concentrations in the final digest, compromising overall functional efficacy. Enhanced prediction of protein-derived bioactivity creates novel opportunities to design multifunctional proteins that retain core functionality while ensuring the release of therapeutically relevant bioactive peptides post-gastrointestinal digestion, thereby enhancing practical utility.

Collectively, advancements in data quality and computational algorithms will propel AI-driven functional peptide discovery toward enhanced performance and broader translational applications.

## CRediT authorship contribution statement

**Xu Liu:** Writing – original draft, Conceptualization. **Feifei Guan:** Writing – review & editing. **Huiying Luo:** Writing – review & editing. **Bin Yao:** Writing – review & editing, Conceptualization. **Jian Tian:** Writing – review & editing, Conceptualization.

## Declaration of competing interest

The authors declare that they have no known competing financial interests or personal relationships that could have appeared to influence the work reported in this paper.

## Data Availability

No data was used for the research described in the article.

## References

[1] Sajid M., Sabika J., Mehvesh M., Priti M., Adil G., Bilal Ahmad A. (2021). Food biopolymers: structural, functional and nutraceutical properties.

[2] Minkiewicz P., Iwaniak A., Darewicz M. (2019). BIOPEP-UWM database of bioactive peptides: current opportunities. Int J Mol Sci.

[3] Samurailatpam S., Amit Kumar R. (2016). Production of bioactive peptides during soybean fermentation and their potential health benefits. Trends Food Sci Technol.

[4] Yu W., Zhang X., Yu Y., Zhao D., Duan S., Szeto I.M.-Y. (2025). Discovery of milk-derived antimicrobial peptides in human milk by DeepMAMP based on peptidomics technology and deep learning method. Food Chem.

[5] Feng Y.-Z., Zhu Q.-F., Xue J., Chen P., Yu Y. (2023). Shining in the dark: the big world of small peptides in plants. aBIOTECH.

[6] Qin D., Bo W., Zheng X., Hao Y., Li B., Zheng J. (2022). DFBP: a comprehensive database of food-derived bioactive peptides for peptidomics research. Bioinformatics.

[7] Chidike Ezeorba TP., Ezugwu A.L., Chukwuma I.F., Anaduaka E.G., Udenigwe C.C. (2024). Health-promoting properties of bioactive proteins and peptides of garlic (*Allium sativum*). Food Chem.

[8] Zhang M., Luo J., Luo C., Zang Y., Zeng Y., Guo H. (2025). Exploration of antioxidant peptides from crocodile (*Crocodylus siamensis*) meat using modern information technology: virtual-screening and antioxidant mechanisms. Food Res Int.

[9] Ma Y., Guo Z., Xia B., Zhang Y., Liu X., Yu Y. (2022). Identification of antimicrobial peptides from the human gut microbiome using deep learning. Nat Biotechnol.

[10] Chang J., Wang H., Su W., He X., Tan M. (2025). Artificial intelligence in food bioactive peptides screening: recent advances and future prospects. Trends Food Sci Technol.

[11] Wang L., Ma M., Yu Z., Du S. (2021). Preparation and identification of antioxidant peptides from cottonseed proteins. Food Chem.

[12] Vidal-Limon A., Aguilar-Toalá J.E., Liceaga A.M. (2022). Integration of molecular docking analysis and molecular dynamics simulations for studying food proteins and bioactive peptides. J Agric Food Chem.

[13] Andresen S.L. (2002). John McCarthy: father of AI. IEEE Intell Syst.

[14] Njirjak M., Žužić L., Babić M., Janković P., Otović E., Kalafatovic D. (2024). Reshaping the discovery of self-assembling peptides with generative AI guided by hybrid deep learning. Nat Mach Intell.

[15] Khatun M.S., Hasan M.M., Kurata H. (2019). PreAIP: computational prediction of anti-inflammatory peptides by integrating multiple complementary features. Front Genet.

[16] Tubiana J., Schneidman-Duhovny D., Wolfson H.J. (2022). ScanNet: an interpretable geometric deep learning model for structure-based protein binding site prediction. Nat Methods.

[17] Lin Z., Akin H., Rao R., Hie B., Zhu Z., Lu W. (2023). Evolutionary-scale prediction of atomic-level protein structure with a language model. Science.

[18] Elnaggar A., Heinzinger M., Dallago C., Rehawi G., Wang Y., Jones L. (2022). ProtTrans: toward understanding the language of life through self-supervised learning. IEEE Trans Pattern Anal Mach Intell.

[19] Cortes C., Vapnik V. (1995). Support-vector networks. Mach Learn.

[20] Liu Y., Wang Y., Zhang J., Liu B., Ma M., Chang J. (2012). Information computing and applications.

[21] Chen T., He T., Benesty M., Khotilovich V., Tang Y., Cho H. (2014).

[22] Lin C., Xiong S., Cui F., Zhang Z., Shi H., Wei L. (2025). Deep learning in antimicrobial peptide prediction. J Chem Inf Model.

[23] Li C., Zou Q., Jia C., Zheng J. (2024). AMPpred-MFA: an interpretable antimicrobial peptide predictor with a stacking architecture, multiple features, and multihead attention. J Chem Inf Model.

[24] Lai B., Xu J. (2022). Accurate protein function prediction via graph attention networks with predicted structure information. Briefings Bioinf.

[25] Chandra A., Tünnermann L., Löfstedt T., Gratz R. (2023). Transformer-based deep learning for predicting protein properties in the life sciences. eLife.

[26] Min J., Rong X., Zhang J., Su R., Wang Y., Qi W. (2024). Computational design of peptide assemblies. J Chem Theor Comput.

[27] Wang J., Dou X., Song J., Lyu Y., Zhu X., Xu L. (2019). Antimicrobial peptides: promising alternatives in the post feeding antibiotic era. Med Res Rev.

[28] Bin Hafeez A., Jiang X., Bergen P.J., Zhu Y. (2021). Antimicrobial peptides: an update on classifications and databases. Int J Mol Sci.

[29] Chen N., Jiang C. (2023). Antimicrobial peptides: structure, mechanism, and modification. Eur J Med Chem.

[30] Hasper H.E., Kramer N.E., Smith J.L., Hillman J.D., Zachariah C., Kuipers O.P. (2006). An alternative bactericidal mechanism of action for lantibiotic peptides that target lipid II. Science.

[31] Le C.-F., Fang C.-M., Sekaran S.D. (2017). Intracellular targeting mechanisms by antimicrobial peptides. Antimicrob Agents Chemother.

[32] Wang J., Torres I.M., Shang M., Al-Armanazi J., Dilawar H., Hettiarachchi D.U. (2024). Direct and pleiotropic effects of antimicrobial peptide transgene integration on reproductive, growth regulating, and non-coding loci in channel catfish (ictalurus punctatus). Agric Commun.

[33] Gawde U., Chakraborty S., Waghu F.H., Barai R.S., Khanderkar A., Indraguru R. (2023). CAMPR4: a database of natural and synthetic antimicrobial peptides. Nucleic Acids Res.

[34] Ma T., Liu Y., Yu B., Sun X., Yao H., Hao C. (2025). DRAMP 4.0: an open-access data repository dedicated to the clinical translation of antimicrobial peptides. Nucleic Acids Res.

[35] Novković M., Simunić J., Bojović V., Tossi A., Juretić D. (2012). DADP: the database of anuran defense peptides. Bioinformatics.

[36] Pirtskhalava M., Amstrong A.A., Grigolava M., Chubinidze M., Alimbarashvili E., Vishnepolsky B. (2021). DBAASP v3: database of antimicrobial/cytotoxic activity and structure of peptides as a resource for development of new therapeutics. Nucleic Acids Res.

[37] Wang G., Li X., Wang Z. (2016). APD3: the antimicrobial peptide database as a tool for research and education. Nucleic Acids Res.

[38] Yao L., Guan J., Xie P., Chung C.-R., Zhao Z., Dong D. (2025). dbAMP 3.0: updated resource of antimicrobial activity and structural annotation of peptides in the post-pandemic era. Nucleic Acids Res.

[39] Usmani S.S., Kumar R., Kumar V., Singh S., Raghava G.P.S. (2018). AntiTbPdb: a knowledgebase of anti-tubercular peptides. Database.

[40] Li C., Sutherland D., Hammond S.A., Yang C., Taho F., Bergman L. (2022). AMPlify: attentive deep learning model for discovery of novel antimicrobial peptides effective against WHO priority pathogens. BMC Genom.

[41] Yan K., Lv H., Guo Y., Peng W., Liu B. (2023). sAMPpred-GAT: prediction of antimicrobial peptide by graph attention network and predicted peptide structure. Bioinformatics.

[42] Murakami Y., Ishida S., Demizu Y., Terayama K. (2023). Design of antimicrobial peptides containing non-proteinogenic amino acids using multi-objective bayesian optimisation. Digital Discovery.

[43] Brizuela C.A., Liu G., Stokes J.M., De La Fuente-Nunez C. (2025). AI methods for antimicrobial peptides: progress and challenges. Microb Biotechnol.

[44] Yan J., Zhang B., Zhou M., Campbell-Valois F.-X., Siu S.W.I. (2023). A deep learning method for predicting the minimum inhibitory concentration of antimicrobial peptides against escherichia coli using multi-branch-CNN and attention. mSystems.

[45] Santos-Júnior C.D., Pan S., Zhao X.-M., Coelho L.P. (2020). Macrel: antimicrobial peptide screening in genomes and metagenomes. PeerJ.

[46] Santos-Júnior C.D., Torres M.D.T., Duan Y., Rodríguez del Río Á., Schmidt T.S.B., Chong H. (2024). Discovery of antimicrobial peptides in the global microbiome with machine learning. Cell.

[47] Huang J., Xu Y., Xue Y., Huang Y., Li X., Chen X. (2023). Identification of potent antimicrobial peptides via a machine-learning pipeline that mines the entire space of peptide sequences. Nat Biomed Eng.

[48] Wang Y., Zhao L., Li Z., Xi Y., Pan Y., Zhao G. (2025). A generative artificial intelligence approach for the discovery of antimicrobial peptides against multidrug-resistant bacteria. Nat Microbiol.

[49] Bergamini C.M., Gambetti S., Dondi A., Cervellati C. (2004). Oxygen, reactive oxygen species and tissue damage. Curr Pharm Des.

[50] Sies H. (2015). Oxidative stress: a concept in redox biology and medicine. Redox Biol.

[51] Hayes J.D., Dinkova-Kostova A.T., Tew K.D. (2020). Oxidative stress in cancer. Cancer Cell.

[52] Liguori I., Russo G., Curcio F., Bulli G., Aran L., Della-Morte D. (2018). Oxidative stress, aging, and diseases. Clin Interv Aging.

[53] Krutmann J., Schikowski T., Morita A., Berneburg M. (2021). Environmentally-induced (extrinsic) skin aging: exposomal factors and underlying mechanisms. J Invest Dermatol.

[54] Sies H., Belousov V.V., Chandel N.S., Davies M.J., Jones D.P., Mann G.E. (2022). Defining roles of specific reactive oxygen species (ROS) in cell biology and physiology. Nat Rev Mol Cell Biol.

[55] César A.P.C., Lopes F.E.S., Azevedo F.F.N., Pinto Y.O., Andrade C.R., Mesquita F.P. (2024). Antioxidant peptides from plants: a review. Phytochem Rev.

[56] Sun J.Z., Kaur H., Halliwell B., Li X.Y., Bolli R. (1993). Use of aromatic hydroxylation of phenylalanine to measure production of hydroxyl radicals after myocardial ischemia in vivo. Direct evidence for a pathogenetic role of the hydroxyl radical in myocardial stunning. Circ Res.

[57] Zhang R., He S., Di D., Li H., Qiu M., Wu Z. (2025). An updated review on soy antioxidant peptides (SAPs): molecular mechanisms, high-tech generation pipeline, industrial production strategies, and new product innovation. Food Rev Int.

[58] Gao J., Li T., Chen D., Gu H., Mao X. (2021). Identification and molecular docking of antioxidant peptides from hemp seed protein hydrolysates. Lebensm Wiss Technol.

[59] Li Q., Shi C., Wang M., Zhou M., Liang M., Zhang T. (2019). Tryptophan residue enhances *in vitro* walnut protein-derived peptides exerting xanthine oxidase inhibition and antioxidant activities. J Funct Foods.

[60] Omoni A.O., Aluko R.E. (2006). Effect of cationic flaxseed protein hydrolysate fractions on the in vitro structure and activity of calmodulin-dependent endothelial nitric oxide synthase. Mol Nutr Food Res.

[61] Vijitpunyaruk T., Theerakulkait C. (2014). Preparation of alcalase hydrolysed rice bran protein concentrate and its inhibitory effect on soybean lipoxygenase activity. Int J Food Sci Technol.

[62] Wang F., Weng Z., Lyu Y., Bao Y., Liu J., Zhang Y. (2020). Wheat germ-derived peptide ADWGGPLPH abolishes high glucose-induced oxidative stress *via* modulation of the PKCζ/AMPK/NOX4 pathway. Food Funct.

[63] Ying F., Lin S., Li J., Zhang X., Chen G. (2021). Identification of monoamine oxidases inhibitory peptides from soybean protein hydrolysate through ultrafiltration purification and *in silico* studies. Food Biosci.

[64] Wong F.-C., Xiao J., Wang S., Ee K.-Y., Chai T.-T. (2020). Advances on the antioxidant peptides from edible plant sources. Trends Food Sci Technol.

[65] Deng W., Chen Y., Sun X., Wang L. (2023). AODB: a comprehensive database for antioxidants including small molecules, peptides and proteins. Food Chem.

[66] Hesamzadeh P., Seif A., Mahmoudzadeh K., Ganjali Koli M., Mostafazadeh A., Nayeri K. (2024). De novo antioxidant peptide design via machine learning and DFT studies. Sci Rep.

[67] Li R., Wang H., Yu Q., Cai J., Jiang L., Luo X. (2025). AOPxSVM: a support vector machine for identifying antioxidant peptides using a block substitution matrix and amino acid composition, transformation, and distribution embeddings. Foods.

[68] Olsen T.H., Yesiltas B., Marin F.I., Pertseva M., García-Moreno P.J., Gregersen S. (2020). AnOxPePred: using deep learning for the prediction of antioxidative properties of peptides. Sci Rep.

[69] Qin D., Jiao L., Wang R., Zhao Y., Hao Y., Liang G. (2023). Prediction of antioxidant peptides using a quantitative structure−activity relationship predictor (AnOxPP) based on bidirectional long short-term memory neural network and interpretable amino acid descriptors. Comput Biol Med.

[70] Lv L., Lv Q., Yang Y., Xiong F., Pei S., He S. (2025). Identification of novel antioxidant peptides from cottonseed meal co-fermented with *lactobacillus mucosae* LLK-XR1 and acid proteases: in silico screening, molecular simulation, and in vitro functional analysis. Food Chem.

[71] Ren L., Fan J., Yang Y., Liu X., Wang B., Bian X. (2023). Identification, in silico selection, and mechanism study of novel antioxidant peptides derived from the rice bran protein hydrolysates. Food Chem.

[72] Chen F., Liu H., Yan J., Shi Q., Yang H., Cao S. (2024). Identification and molecular mechanism of novel antioxidant peptides from squid skin protein hydrolysates: in silico and in vitro analysis. Lebensm Wiss Technol.

[73] Zhu L., Xiong H., Huang X., Guyonnet V., Ma M., Chen X. (2022). Identification and molecular mechanisms of novel antioxidant peptides from two sources of eggshell membrane hydrolysates showing cytoprotection against oxidative stress: a combined in silico and in vitro study. Food Res Int.

[74] Xiao C., Toldrá F., Zhao M., Zhou F., Luo D., Jia R. (2022). In vitro and in silico analysis of potential antioxidant peptides obtained from chicken hydrolysate produced using alcalase. Food Res Int.

[75] Dadar M., Shahali Y., Chakraborty S., Prasad M., Tahoori F., Tiwari R. (2019). Antiinflammatory peptides: current knowledge and promising prospects. Inflamm Res.

[76] Lawrence T. (2009). The nuclear factor NF-κB pathway in inflammation. Cold Spring Harbor Perspect Biol.

[77] Chen L., Deng H., Cui H., Fang J., Zuo Z., Deng J. (2017). Inflammatory responses and inflammation-associated diseases in organs. Oncotarget.

[78] de Medeiros A.F., de Queiroz J.L.C., Maciel B.L.L., de Araújo Morais A.H. (2022). Hydrolyzed proteins and vegetable peptides: anti-inflammatory mechanisms in obesity and potential therapeutic targets. Nutrients.

[79] Kwak S.-J., Kim C.-S., Choi M.-S., Park T., Sung M.-K., Yun J.W. (2016). The soy peptide phe–leu–val reduces TNFα-induced inflammatory response and insulin resistance in adipocytes. J Med Food.

[80] Liang Q., Chalamaiah M., Liao W., Ren X., Ma H., Wu J. (2020). Zein hydrolysate and its peptides exert anti-inflammatory activity on endothelial cells by preventing TNF-α-induced NF-κB activation. J Funct Foods.

[81] Paz SM la, Lemus-Conejo A., Toscano R., Pedroche J., Millan F., Millan-Linares M.C. (2019). GPETAFLR, an octapeptide isolated from Lupinus angustifolius L. protein hydrolysate, promotes the skewing to the M2 phenotype in human primary monocytes. Food Funct.

[82] Yi G., Li H., Liu M., Ying Z., Zhang J., Liu X. (2020). Soybean protein-derived peptides inhibit inflammation in LPS-induced RAW264.7 macrophages via the suppression of TLR4-mediated MAPK-JNK and NF-kappa B activation. J Food Biochem.

[83] Vita R., Blazeska N., Marrama D., Duesing S., Bennett J. (2025). The immune epitope database (IEDB): 2024 update. Nucleic Acids Res.

[84] Gupta S., Sharma A.K., Shastri V., Madhu M.K., Sharma V.K. (2017). Prediction of anti-inflammatory proteins/peptides: an insilico approach. J Transl Med.

[85] Manavalan B., Shin T.H., Kim M.O., Lee G. (2018). AIPpred: sequence-based prediction of anti-inflammatory peptides using random forest. Front Pharmacol.

[86] Zhao D., Teng Z., Li Y., Chen D. (2021). iAIPs: identifying anti-inflammatory peptides using random forest. Front Genet.

[87] Liu R., Fu X., Yan S., Zhang Z., Cui F. (2023). 2023 IEEE International Conference on Bioinformatics and Biomedicine (BIBM).

[88] Gaffar S., Hassan M.T., Tayara H., Chong K.T. (2024). IF-AIP: a machine learning method for the identification of anti-inflammatory peptides using multi-feature fusion strategy. Comput Biol Med.

[89] Zhu L., Yang Q., Yang S. (2024). DeepAIP: deep learning for anti-inflammatory peptide prediction using pre-trained protein language model features based on contextual self-attention network. Int J Biol Macromol.

[90] Aluko R.E. (2015). Antihypertensive peptides from food proteins. Annu Rev Food Sci Technol.

[91] Bao X., Zhang Y., Wang L., Dai Z., Zhu Y., Huo M. (2025). Machine learning discovery of novel antihypertensive peptides from highland barley protein inhibiting angiotensin I-converting enzyme (ACE). Food Res Int.

[92] Jiang S., Mo F., Li W., Yang S., Li C., Jiang L. (2025). Deep learning-driven optimization of antihypertensive properties from whey protein hydrolysates: a multienzyme approach. J Agric Food Chem.

[93] Hong Z., Cheng Y., Zhou M., Wang S., Li X., Feng S. (2025). Exploring anxiolytic peptides derived from walnut protein by an integrated approach of multivariate analysis, random forest methodology. Food Biosci.

[94] Hafeez Z., Benoit S., Cakir-Kiefer C., Dary A., Miclo L. (2021). Food protein-derived anxiolytic peptides: their potential role in anxiety management.

[95] Ashaolu T.J., Zarei M., Agrawal H., Kharazmi M.S., Jafari S.M. (2024). A critical review on immunomodulatory peptides from plant sources; action mechanisms and recent advances. Crit Rev Food Sci Nutr.

[96] Hwang J.S., Kim S.G., Shin T.H., Jang Y.E., Kwon D.H., Lee G. (2022). Development of anticancer peptides using artificial intelligence and combinational therapy for cancer therapeutics. Pharmaceutics.

[97] Datta S., Yu J.-C., Lin Y.-H., Cheng Y.-C., Chen C.-T. (2025). AAGP integrates physicochemical and compositional features for machine learning-based prediction of anti-aging peptides. Sci Rep.

[98] Li X.-H., Su W.-R., Wang F.-F., Li K., Zhu J.-Y., Zhu S.-Y. (2023). Computational biology in topical bioactive peptide discovery for cosmeceutical application: a concise review. Biomed Eng Commun.

[99] Chen X., Huang J., He B. (2022). AntiDMPpred: a web service for identifying anti-diabetic peptides. PeerJ.

[100] Peng C.-X., Zhou X.-G., Xia Y.-H., Zhang Y., Zhang G.-J. (2021).

[101] D'Andrea L.D., Romanelli A. (2023). Temporins: multifunctional peptides from frog skin. Int J Mol Sci.

[102] Tian X., Chen C., Cyr K., Dong G., Salim H., Li Y. (2017). Targeted killing of *Streptococcus* mutans in biofilms by a pheromone guided antimicrobial peptide HP30. J Antibiot Res.

[103] Wei X., Wu R., Zhang L., Ahmad B., Si D., Zhang R. (2018). Expression, purification, and characterization of a novel hybrid peptide with potent antibacterial activity. Molecules.

[104] Zhang Y., Liu Y., Li W., Han W. (2024). Antioxidant activity of soybean peptides and Keap1 protein: a combined in vitro and in silico analysis. Lebensm Wiss Technol.

[105] Juretić D. (2022). Designed multifunctional peptides for intracellular targets. Antibiotics.

[106] Kang Y., Zhang H., Wang X., Yang Y., Jia Q. (2024). MMDB: multimodal dual-branch model for multi-functional bioactive peptide prediction. Anal Biochem.

[107] Tang W., Dai R., Yan W., Zhang W., Bin Y., Xia E. (2022). Identifying multi-functional bioactive peptide functions using multi-label deep learning. Briefings Bioinf.

[108] Gao H., Guan F., Luo B., Zhang D., Liu W., Shen Y. (2025). DLFea4AMPGen de novo design of antimicrobial peptides by integrating features learned from deep learning models. Nat Commun.

[109] Du Z., Ding X., Xu Y., Li Y. (2023). UniDL4BioPep: a universal deep learning architecture for binary classification in peptide bioactivity. Briefings Bioinf.

[110] Purohit K., Reddy N., Sunna A. (2024). Exploring the potential of bioactive peptides: from natural sources to therapeutics. Int J Mol Sci.

[111] Zhao A., Wu Y., Yue Y., Wu T., Xu Q., Yue Y. (2025).

[112] Kang Z., Wang Z., Wang J., Liu Q., Pan D., Wu Z. (2025). Production of bioactive peptides by high-voltage pulsed electric field: protein extraction, mechanism, research status and collaborative application. Food Chem.

